# Detection of water-molecular-motion configuration in patients with lupus nephritis: a primary study using diffusion-weighted imaging

**DOI:** 10.1186/s12882-020-01955-x

**Published:** 2020-07-29

**Authors:** Huilan Shi, Yanyan Wang, Tiekun Yan, Junya Jia, Dong Li, Li Wei, Wenya Shang, Zhenfeng Zheng

**Affiliations:** 1 Department of Radiology, Tianjin Medical University General Hospital, Tianjin, China; 2 Department of Nephrology, Tianjin Medical University General Hospital, No.154 Anshan Road, Heping District, Tianjin, China

**Keywords:** Lupus nephritis, Diffusion-weighted imaging, Apparent diffusion coefficient, Repeated-measures analysis of variance

## Abstract

**Background:**

Lupus nephritis (LN) is one of most common types of secondary glomerulonephritis, which is characterized by longitudinal pathological changes. Microstructural lesions of LN will impact the motion of water molecules, which can be detected by diffusion-weighted imaging (DWI). There are few reported measurements of water diffusion in patients with LN, and the nature of water diffusion across the entire depth of the renal parenchyma remains largely unknown.

**Methods:**

Twenty adult patients with LN and 11 healthy volunteers underwent DWI inspection. Renal biopsy samples were characterized based on the revised ISN/RPS 2003 classification. The apparent-diffusion coefficient (ADC) was calculated via fitting into a mono-exponential model. To compare the ADC level across the entire renal parenchyma between the two groups, repeated-measures analysis of variance (RM-ANOVA) was performed. ADC data derived from DWI pictures were transformed into tridimensional maps by MATLAB software.

**Results:**

Compared with data from healthy volunteers, lower average ADC values with major undulatory magnitudes were found in patients with LN, especially in the cortical zone. Tridimensional maps of patients with LN displayed geographic terrain-like canyons and/or valleys that were different from the corresponding terrain-like flatlands and/or plateaus in healthy volunteers. A heterogeneity of ADC values was found in bilateral kidneys. Left kidneys predominated higher ADC values in patients with LN. The ADC values across the entire renal parenchyma exhibited statistically significant differences among the three identified pathological subclasses (*P* < 0.001).

**Conclusions:**

Analysis of the motion of water molecules across the entire renal parenchyma may be helpful for better understanding the pathological conditions of LN, for which microstructural and functional heterogeneity may be detected and visualized via DWI.

## Background

Systemic lupus erythematosus (SLE) is an autoimmune disease that is characterized by immunological dysfunction of aberrant autoantibody production and potential multiorgan involvement [1]. Lupus nephritis (LN) is recognized as one of the most severe complications. An epidemiological survey showed that LN is one of the most common types of secondary glomerulonephritis in China [2]. A retrospective cohort study revealed that compared with that of the general population, LN patients have a six-fold higher rate of mortality. When LN patients develop to end-stage renal disease (ESRD), the corresponding mortality rate increases by 26-fold, which is more than twice the risk associated with malignancy or cardiovascular disease [3]. Precise clinical diagnoses and comprehensive pathophysiological evaluations are crucial for nephrologists to identify and treat LN patients. Although renal biopsies have played a critical role in diagnoses for many years, this invasive type of inspection has become restricted due to its high risk of bleeding, especially for those with coagulant abnormalities or renal atrophy [4]. Moreover, the approach of detecting changes in the histological pattern of LN is problematic. Lu et al. reviewed the pathological documents of 156 LN patients with repeat renal biopsies, of which the percentage of histological transformation was only 75% [5]. Hence, nephrologists need a novel, noninvasive, and repeatable inspection approach to better identify and understand the renal pathological conditions of LN.

Diffusion-weighted imaging (DWI), which is a functional magnetic resonance imaging (fMRI) technique used for detecting the free random motion of water molecules within tissues, has been shown to have considerable value for the evaluation of pathological conditions and for the resolution of the microstructure of renal tissues [6, 7]. Previous studies have focused on the assessment of renal function [8] and renal interstitial fibrosis [9]. Our research team has found that proliferative lesions that impact renal microstructure can be detected by parameters from DWI [10]. These findings demonstrate that DWI is particularly sensitive to alterations in the renal interstitium—including fibrosis, cellular proliferation and infiltration—in terms of renal-tissue microcirculation and water restricted within the tubular compartment. By analyzing renal DWI data, we have an opportunity to better understand renal microscopic changes. In principal, the DWI signal from biological tissues decays proportionally to the strength of the magnetic gradient applied [11]. The first widely accepted water-molecular-motion model is the monoexponential model, which assumes that water diffusion adheres to a Gaussian distribution. In this framework, a diffusion coefficient can be used as a quantitative index for describing the motion of water molecules. Since the molecular motion of water is not free but is hindered by many obstacles, including cell membranes and interstitial matrices, the apparent diffusion coefficient (ADC) has been used for data from biological tissues to account for these influences. Although there have been more published investigations regarding the correlation of ADC values with renal pathological changes in recent years [12, 13], such studies focusing on LN patients have been limited. To the best of our best knowledge, aside from our previous study [10], only three similar studies have been published [12, 14, 15].

The aim of this study was to investigate the configuration of water molecular diffusion in patients with LN by visualizing and analyzing ADC data of the renal parenchyma. The heterogeneity of renal ADC values in bilateral kidneys was also analyzed. In addition, we investigated the range of ADC values, from superficial cortex to the inner medulla, in terms of identifying pathological subclasses of LN.

## Methods

### Study protocol

#### Patients

Our clinical study was beginning from January 2017 and finished at June 2018. A total of thirty-one participants were finally recruited including twenty patients with lupus nephritis and eleven healthy volunteers. Our study protocol had been approved by local Ethical Committee before our investigation was put into practice. Patients with lupus nephritis was diagnosed by experienced nephrologists, who had consulted the systemic lupus erythematosus 2012 International Collaborating Clinics classification criteria [16]. Renal filtration function was evaluated by calculating the estimated glomerular filtration rate (eGFR) [17]. Clinical activity of SLE was also assessed by SLE disease activity index (SLEDAI) [18]. Detailed information of patients with lupus nephritis and healthy volunteers were showed in supplementary Table 1 and Table 2. Since renal oxygenation conditions were influenced by numerous factors, such as oxygen supplementation and oxygen consumption, several special conditions should be avoided as much as possible. These conditions included oxygen inhalation, water or slat overload, specific medication such as diuretics, vasodilators or hematopoietin. We set 7–10 days period to cease some medicine which could potentially affect DWI imaging. We also required that the daily fluctuation of body weight did not exceeded 2.5%. Subjects fasted and did not drink water for at least 8 h before MRI examination.

### Renal histopathology

Renal histopathological diagnosis was based on historical manifestation via light microscopy. Four histological staining including hematoxylin and eosin, periodic-acid Schiff, silver methenamine, and Masson’s trichrome have been used to visualise pathological changes. Two experienced pathologists independently provided pathological diagnostic results according to the revised ISN/RPS 2003 classification [19]. Disagreement was resolved by consensus or with a third pathologist. Renal biopsy samples with less than 10 glomeruli were excluded. Primary classification was adopted as renal diagnosis. For example, cases of IV + V were classified as class IV. Renal pathological activity assessment was adopted a semi-quantitative scoring system [20]. .

### MRI protocol

MRI was performed using a 3.0-T Imager (GE Discovery 750 3.0 T, General Electric, USA). The detailed imaging parameters can be referred to our previous study [10].

### Image analysis

**Fig. 1:**
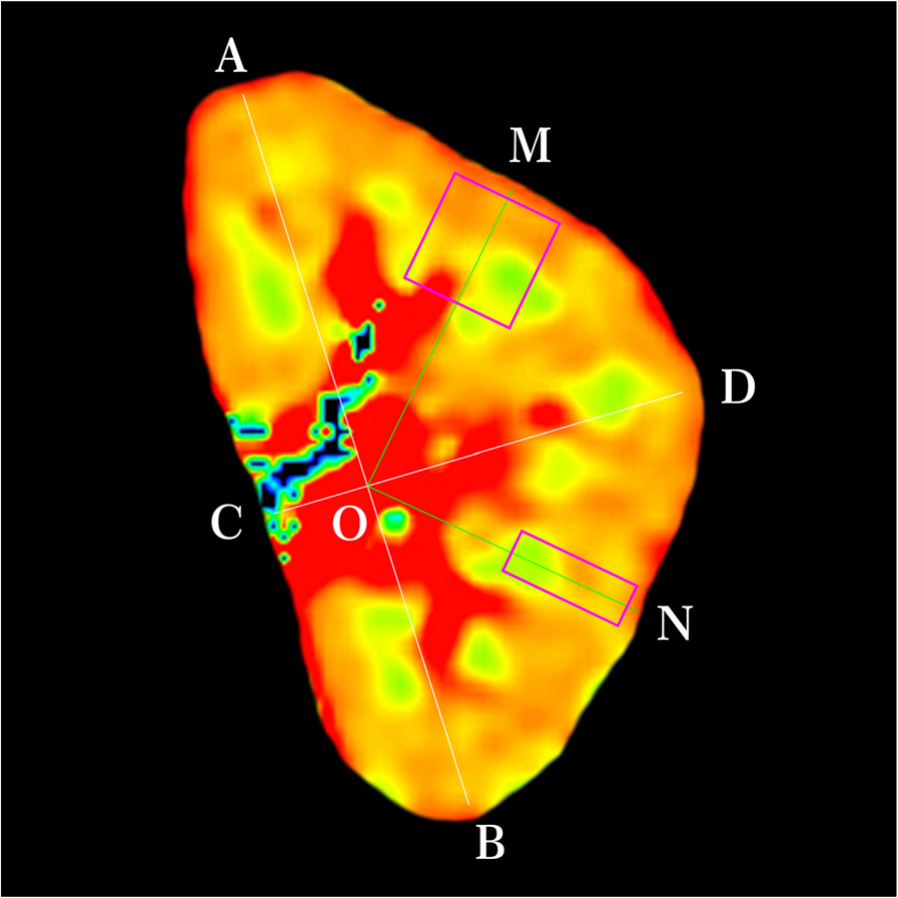
Establishment of ROIs for the renal DWI map. This coronal section of the renal DWI map came from the right kidney of a patient with LN. The line **CD** linked the renal middle pole and the renal hilus. The line **AB** was connected between the renal upper pole and the lower pole. Point **O** was the junction of the two lines. In order to acquire consecutive ADC data along the direction from the superficial cortex to the deep layer of the medulla, the rectangular ROI and squared ROI were placed in the renal-parenchyma BOLD image. One tip of the ROI was located on the renal-cortical surface. Another tip of the ROI was oriented toward the point **O**. The long axes of the rectangular ROI and squared ROI are shown by the green lines

ADC maps were constructed on an ADW 4.5 Workstation using the FUNCTOOL program. The largest renal coronal-anatomical plane was selected in each kidney. The entire kidney section, including both cortex and medulla, was selected; the renal-collecting system and any incidental renal cysts were excluded. We set two kinds of regions of interest (ROIs). One ROI was a rectangular ROI that was set as a 20-pixel (width) × 50-pixel (height) rectangle. The other ROI was a squared ROI that was set as a 50-pixel (width) × 50-pixel (height) square. One tip of the ROI was located on the renal cortical surface. Another tip of the ROI was oriented toward the renal hilus (Fig. 1). According to the standard ROI setting protocol, all of multiple ROI samples were obtained by three independent radiologists who were blinded each other. ADC values of each voxel of the selected ROI were obtained with MATLAB 2014a (MathWorks Inc., Natick, MA, USA).

The ADC value was calculated using a mono-exponential function. The relationship between signal variation and *b* factors was expressed using the following equation:

$$ {S}_b/{S}_0={e}^{- bADC} $$

### Statistical analyses

#### RM-ANOVA data

ADC values of renal BOLD maps were expressed as mean ± standard deviation (SD) and mean ± standard error of means (−SEM). To compare the ADC level difference between the LN group and the control group at different depths of the renal parenchyma, repeated-measures analysis of variance (RM-ANOVA) was performed. Mauchly’s test was used to check whether the covariance structure satisfied the sphericity condition [21]. If the covariance matrix satisfied the sphericity assumption, the univariate RM-ANOVA was applied for further analysis. Otherwise, a multivariate ANOVA was used to analyze the data [22], and four multivariate test statistics were then calculated: Pillai’s Trace statistic, Wilks’ likelihood ratio, the Hotelling-Lawley Trace criterion, and Roy’s Largest Root. The effects of intergroup (LN vs healthy control) and intragroup (different depths of renal parenchyma) factors were compared. The interaction effect was also compared. Statistical significance was accepted at *P* < 0.05. All analyses were carried out using the IBM® SPSS® Statistics software (version 22.0.0.0 IBM Corporation, Armonk, NY, USA).

## Results

### Demographic information of participants

There were twenty patients in the LN group who were included in the study and underwent DWI-MRI as well as renal biopsy inspections. Eleven healthy volunteers who received DWI-MRI inspections were also involved. The healthy volunteers who were included in our study were recruited from different divisions of our hospital. The gender ratios between the two groups were similar, although female participants were predominant in both groups. Detailed demographic information of participants was listed in Table 1 and Supplementary Table 1 and Table 2.

**Table 1:** Demographic information of participants

Participants parameters	Values (mean ± SD)
Patients with lupus nephritis (*N* = 20)	
Age (years)	35.00 ± 13.97
Female/male ratio	20/2
Urine protein (g/day)	3.85 ± 2.72
eGFR (ml/min/1.73 m^2^)	99.40 ± 25.60
Pathological patterns	
III-(A/C)	2
III-(A/C) + V	3
IV-G(A/C)	3
IV-S(A/C)	1
IV-S(A)	1
IV-G(A/C) + V	5
IV-S(A/C) + V	1
V	4
Healthy volunteers (*N* = 11)	
Age (years)	33.00 ± 10.87
Female/male ratio	11/1
eGFR (ml/min/1.73 m^2^)	115.82 ± 14.08
Source of healthy volunteers	
Physical-examination center	6
Division of general surgery	2
Division of endocrinology	1
Division of ophthalmology	1
Division of dermatology	1

### ADC values of the renal parenchyma between LN patients and healthy volunteers

**Fig. 2:**
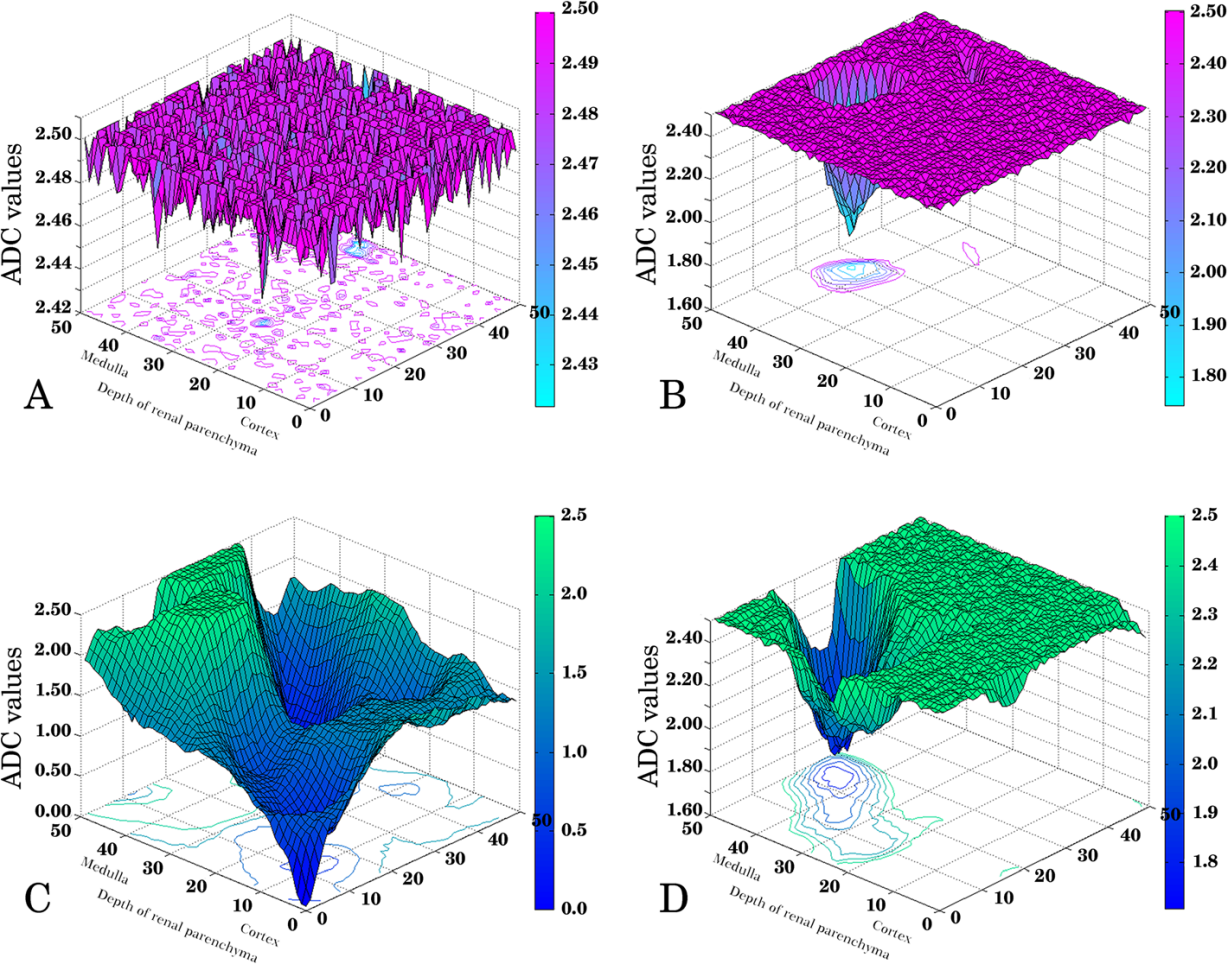
Configuration of renal-parenchyma ADC values in LN patients and healthy volunteers. Four samples of ADC values from one healthy volunteer (**a** and **b**) and one patient with LN (**c** and **d**) are shown. In the healthy volunteer, the majority of ADC values in the renal parenchyma fluctuated with a slight magnitude (Panel **a**). Sporadically lower ADC values of regions scattered in the renal parenchyma, especially in the medullary zone (Panel **b**). In the LN patient, a broad region of renal tissue displayed attenuated ADC values, even in the cortical zone (Panel **c**). A heterogeneity of ADC values was readily detected in renal tissue (Panel **d**)

We studied the ROI samples for both LN patients and healthy volunteers and visualized ADC data of ROI samples by MATLAB software. In the healthy-volunteers group, renal-parenchyma ADC data exhibited principal features. One feature was that there were uniformly high ADC values in the majority of regions within kidney tissue. Renal-tissue ADC values fluctuated slightly in magnitude, especially in the cortical zone. Another feature was that there were localized lower ADC-value regions scattered sporadically in the renal parenchyma, particularly in the medullary zone. In contrast, disparate characteristics of ADC values existed in LN patients. Not only were the average ADC values lower than those in healthy volunteers, but there were also lower ADC-value regions that were widely distributed in the renal parenchyma, even in the cortical zone. Figure 2 shows the distribution of renal ADC values in one LN patient and in one healthy volunteer.

### Heterogeneity of ADC configuration between bilateral kidneys

**Fig. 3:**
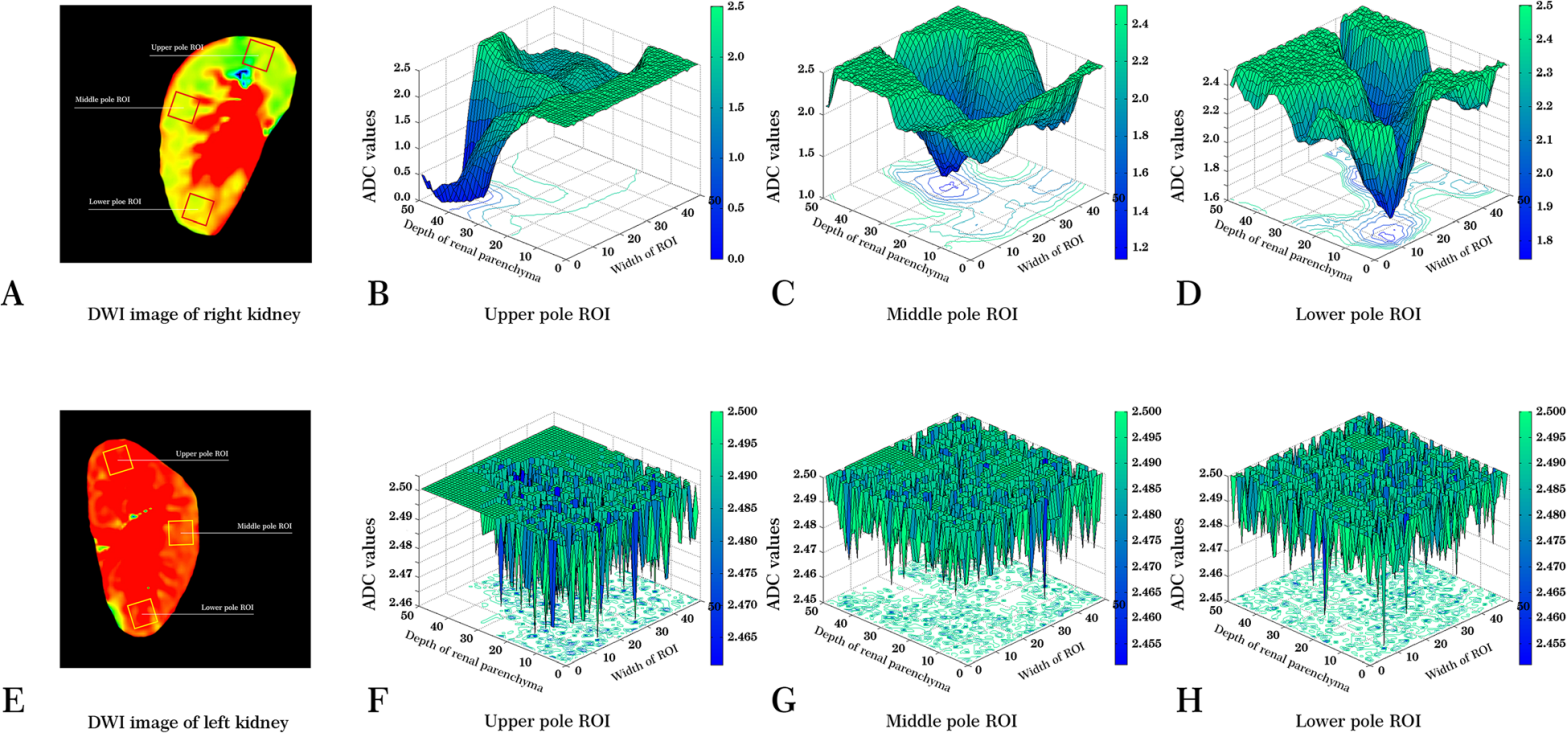
Manifestation of bilateral-renal ADC values in one LN patient. A heterogeneity of ADC values was observed in LN patients. This picture shows the distinctly different renal ADC values in a 46-year-old female patient with type-IV LN. Two coronal-section DWI images were acquired from bilateral kidneys. Three squared ROIs were placed at the renal upper, middle and lower pole in a unilateral kidney. Panels **a** and **e**. ADC values in each squared ROI were visualized in three-dimensional images by MATLAB software. Since obviously irregular and abnormal shapes of ADC values were found in the left kidney, it suggests that the pathophysiological injuries in the left kidney were more comparatively more serious (Panels **b**, **c** and **d**). In contrast, more regular distributions of ADC values could be seen in the right kidney, and we speculated that slight injuries might exist in right kidney (Panels **f**, **g** and **h**)

Our study also compared the difference in ADC values between the left kidney and right kidney within the same subject. In the healthy-volunteers group, six subjects showed higher renal-parenchyma ADC values in their right kidneys and only one subject higher ADC values in the left kidney. The other four subjects exhibited no differences in the ADC values between their two kidneys. However, a disparate trend was found in LN patients. Aside from eight LN patients who had similar ADC values in their bilateral kidneys, the majority of LN patients (nine cases) exhibited higher ADC values in their left kidneys. Only three LN patients had lower ADC values in their left kidneys. Figure 3 shows the heterogeneity of ADC configurations across the bilateral kidneys.

### Distribution of renal ADC values from the superficial-cortex zone to inner-medullary zone

To investigate the distribution of ADC values across the depth of the renal parenchyma, we compared the ADC data at different depths of the renal parenchyma between LN patients and healthy volunteers via an RM-ANOVA. Since Mauchly’s test showed that the covariance structure was not satisfied with the sphericity assumption (*P* < 0.001), a multivariate ANOVA was chosen for further analysis. Statistical differences in ADC values between renal-parenchyma layers were observed in both the LN group and the healthy-volunteer group (Pillai’s Trace statistic, *P <* 0.001; Wilks’ likelihood ratio, *P <* 0.001; Hotelling-Lawley Trace criterion, *P <* 0.001; Roy’s Largest Root, *P <* 0.001). A statistically significant interaction between renal-parenchyma layers and study groups was also observed (Pillai’s Trace statistic, *P* < 0.001; Wilks’ likelihood ratio, *P* < 0.001; Hotelling-Lawley Trace criterion, *P* < 0.001; Roy’s Largest Root, *P* < 0.001). These statistical results indicate that differences in the renal ADC configuration existed not only across the depth of the renal parenchyma within individuals, but that this parameter was also different across the two experimental groups.

**Fig. 4:**
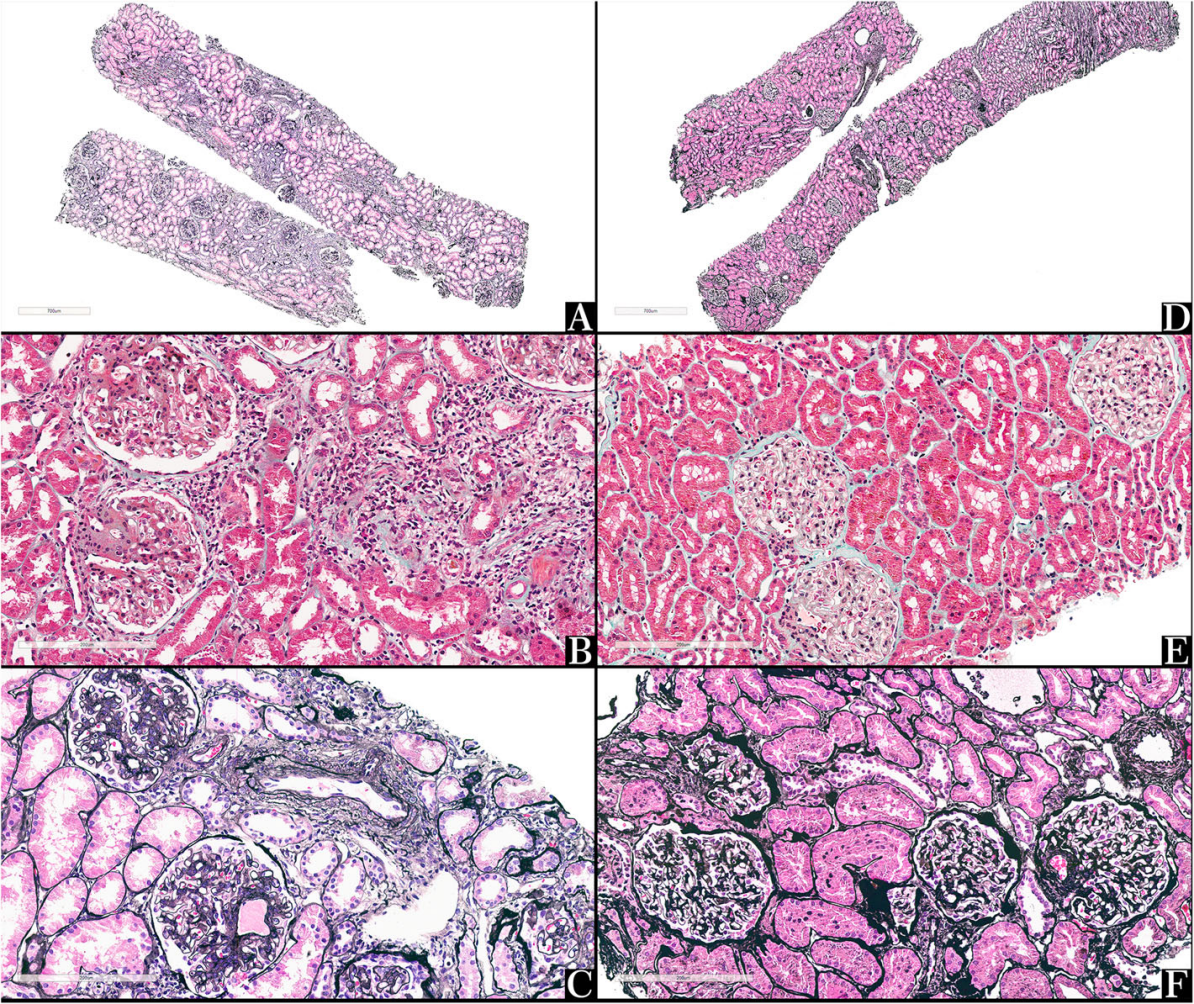
Differences of renal pathological manifestations in type-IV and type-V LN patients. Renal-biopsy samples showed in the pictures came from two patients with LN, whose pathological patterns were type IV for one patient and type V for the other patient. One patient was a 48-year-old male patient with a IV-S(A/C) subclass according to the ISN/RPS 2003 classification (Panels **a**, **b** and **c**). **a** Panoramic view of the renal-biopsy sample. Focal or sporadic distribution of tubulointerstitial changes. Jones-methenamine silver staining is shown (× 40). **b** Mesangial-cell proliferation and interstitial inflammatory-cell infiltration are shown. Masson’s trichrome stain was used (× 200). **c** Mesangial-cell and extracellular-matrix proliferation are shown. Jones-methenamine silver staining is shown (× 200). Another example shows a 30-year-old female patient with type-V subclass LN (Panels **d**, **e** and **f**). **d** Panoramic view of the renal-biopsy sample. Minimal changes of the renal tubular area and interstitium. Jones-methenamine silver staining is shown (× 40). **e** Non-proliferative changes in mesangial and endothelial cells are shown. Masson’s trichrome stain, is shown (× 200). **f** The uniform-thickened glomerular-basement membrane is shown. Jones-methenamine silver staining is shown (× 200)

**Fig. 5:**
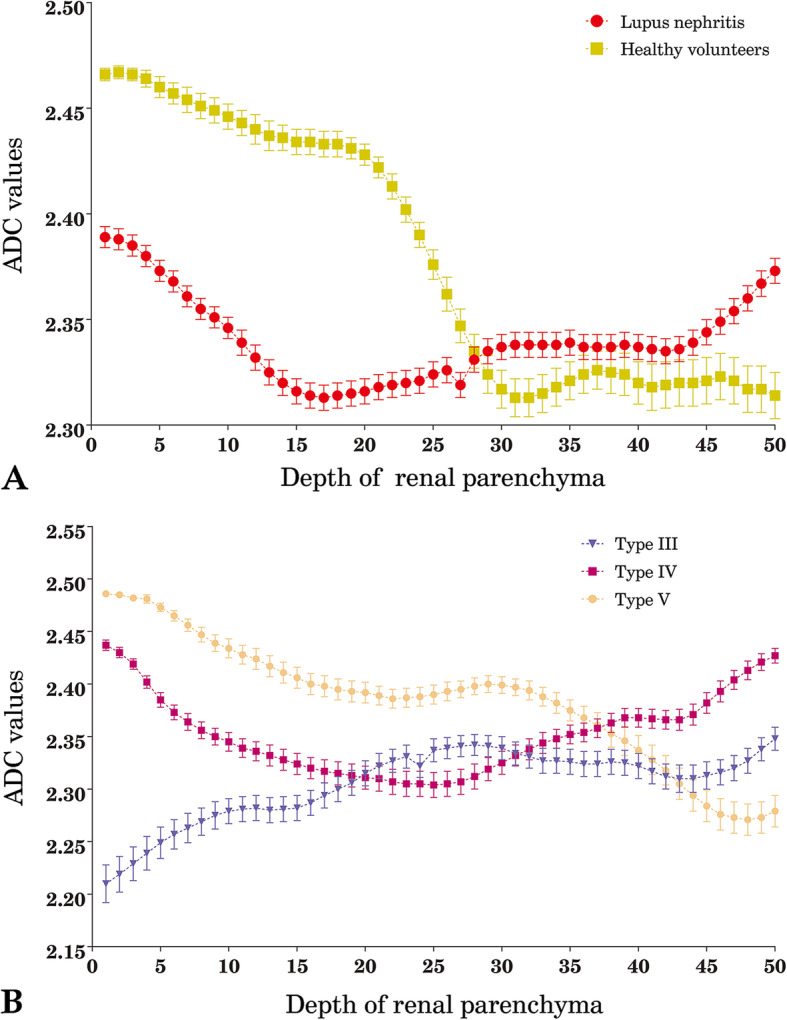
Distribution of renal ADC values from the superficial-cortex zone to the inner-medullary zone. We detected and compared the entire renal-parenchyma ADC values in both LN patients and healthy volunteers. Cherry-red circles and chartreuse squares indicate ADC values for patients with LN and healthy volunteers, respectively. The ADC values in LN patients were lower than those in healthy volunteers, especially in the superficial-cortical zone. However, this tendency was reversed in the deep-medullary zone (Panel **a**). Blue-resupinate triangles, mauve squares and stone-yellow circles stands denote type-III, type-IV and type-V LN, respectively. Type-III LN patients had the lowest cortical ADC values. Subsequently, ADC values were slightly increased in the deep-medullary zone. Accompanied by the increment of depth of the renal parenchyma, ADC values in type-IV LN patients exhibited a V-like or U-like shape. In contrast, the distribution of renal ADC values in type-V LN patients had a similar shape with that of healthy volunteers (Panel **b**). All of data in figure expressed as mean ± SEM

We also investigated the ADC data from different pathological subclasses of LN patients. Similar results were found among the type-III, type-IV and type-V subgroups. There was a statistic difference in the ADC configuration of the three subgroups of LN patients (Pillai’s Trace statistic, *P* < 0.001; Wilks’ likelihood ratio, *P <* 0.001; Hotelling-Lawley Trace criterion, *P <* 0.001; Roy’s Largest Root, *P <* 0.001). Significant differences in the ADC values were also observed in renal-parenchyma layers (Pillai’s Trace statistic, *P <* 0.001; Wilks’ likelihood ratio, *P <* 0.001; Hotelling-Lawley Trace criterion, *P <* 0.001; Roy’s Largest Root, *P <* 0.001; Fig. 4). We also explored the renal ADC configuration in LN patients with type-IV subclass. Three patients whose eGFR were less than 80 ml/min/1.73m^2^ were analyzed with their ADC values of renal parenchyma. We compared renal ADC values between patients with lower eGFR (less than 80 ml/min/1.73m^2^) and patients with normal eGFR (more than 80 ml/min/1.73m^2^) in type-IV subclass. There was a statistic difference in the ADC configuration of two cluster patients (Pillai’s Trace statistic, *P <* 0.001; Wilks’ likelihood ratio, *P <* 0.001; Hotelling-Lawley Trace criterion, *P <* 0.001; Roy’s Largest Root, *P <* 0.001). See Figure Supplement. Figure 5 showed the pathological display between type IV and type V renal biopsy samples.

## Discussion

Our current investigation focused on pathophysiological changes of renal tissue in patients with LN via DWI-MRI. Distinct from the previous related studies, our study focused on the features and distributions of water molecular diffusion rather than merely assessing average levels. After we analyzed ADC data from renal DWI images, two principal results were found. First, conspicuous changes of ADC values existed in the renal cortical zone. ADC values of the renal parenchyma in LN patients were significantly lower than those in healthy volunteers. Moreover, ADC data from LN patients fluctuated more drastically; the corresponding ADC data from healthy volunteers exhibited more homogeneous values with smaller fluctuations in magnitude. By reconstructing ADC data of ROIs into tridimensional maps that were akin to geographical maps, distinctive ADC features were revealed. In the LN group, the majority of the cortical-zone portions of the tridimensional ADC maps displayed a geographic terrain that was canyon-like and/or valley-like. In contrast, the cortical-zone geographical ADC maps from the healthy-volunteers group were flatland-like and/or plateau-like. Secondly, another discovery of our study was the heterogeneity of impaired water molecular motion in LN. By comparing bilateral renal ADC values and configurations of a total of 21 patients, we found that twelve patients showed significantly different ADC values across their two bilateral kidneys. The tridimensional geographic maps also displayed distinctive non-analogous terrain on both sides of the kidneys. To our surprise, the pattern of different ADC values across the two kidneys within the same individual could also be found in healthy volunteers. Specifically, the water molecular motion in left kidneys was slightly greater than that in right kidneys.

Although the precise mechanism underlying the reduced ADC values and their abnormal distributions in LN patients was not elucidated in this study, we speculate that pathophysiological intracellular and/or intercellular changes in renal tissue might impact the motion of water molecules. Theoretically, several categories of proliferative lesions can be found in renal tissues. These lesions include proliferation of endothelial and mesangial cells, proliferation of parietal epithelial cells, cellular or fibrotic crescent formation, and interstitial fibrosis [23]. Since increased cellular densities and extracellular matrix present similar barriers that obstruct the free movement of water, lower ADC values may be detected by DWI-MRI. For example, Li and colleagues detected lower ADC values in LN patients, especially in proliferative LN such as type-III and type-IV subclasses [15]. Another previous study also corroborated this finding. Li et al. investigated the relationship of renal ADC values and renal pathological scores in 71 patients with CKD who had been inspected by renal biopsy. They found that higher renal pathological scores correlated with lower renal ADC values [7]. Whether type-III or type-IV LN subclasses or higher pathological scores were examined, proliferative lesions were the prominent characteristics in the renal tissue.

A functional asymmetry of bilateral kidneys has been documented by nephrologists for many years. Oh et al. detected the glomerular filtration rate (GFR) in each side of the kidney using technetium-99 m diethylenetriaminepentaacetic acid (99mTcDPTA). The left kidneys showed greater renal function, and the functional ratio of the left kidney and the right kidney was 52.5 and 47.5%, respectively [24]. Another related finding came from data of living-donor nephrectomies. Hsu et al. reviewed a large sample of donor nephrectomies comprising 27,942 cases of laparoscopic donor nephrectomies (LDNs) and 8048 cases of open-donor nephrectomies (ODNs). They found that left-sided donor nephrectomies were associated with a lower risk of allograft failure, regardless of open or laparoscopic approaches [25]. Our current study found obvious heterogeneity of ADC configurations in both healthy volunteers and LN patients. To our surprise, the discrepancy of bilateral-renal ADC values in healthy volunteers was opposite to the pattern found in those in LN patients. At present, there are no published studies to explain our findings because relevant asymmetrical studies of bilateral kidneys are lacking. However, we speculate that there was slight asymmetry of renal water molecular diffusion or microcirculation perfusion in the bilateral kidneys. In general, right kidneys may have a superior ability to deliver or distribute liquids compared to left kidneys under healthy physiological conditions. Since autoantibodies and pathogenic cytokines are principal components of the pathogenesis of LN, these autoantibodies or cytokines may be preferentially transported into right kidneys on the basis of left-right asymmetry. Subsequently, proliferative lesions may be more serious in right kidneys and subsequently give rise to decreased ADC values.

Our study also investigated discrepancies in ADC values among the three subgroups of LN patients. ADC values in the type-V subclass were the highest among the three subgroups. Theoretically, lower renal-cortical ADC values should be detected in type-IV-subclass patients with LN. To our surprise, ADC values of the cortical zone in the type-IV subclass were significantly higher than those in the type-III subclass, although pathological injuries in the type-IV subclass were more serious than those in the type-III subclass. According to previous studies, renal-parenchyma ADC values are closely correlated with the magnitude of proliferative lesions. For example. Xu et al. explored the relationship between renal ADC values and histopathologic changes in 52 patients with CKD. They found negative correlations between renal ADC values and scores of tubulointerstitial lesions and severity of interstitial fibrosis [13]. Inoue et al. detected the ADC values in 142 patients with either diabetic nephropathy, CKD without diabetes, or acute-kidney injury. They found that increased fibrosis was significantly correlated with ADC values [26]. As such, it is unclear why lower ADC values were measured in type-III-subclass LN patients in our present study. This surprising result could be due to different constituents of intercellular and extracellular matrices between these two subclasses of LN patients. These renal tissue matrices have different capacities for impacting the molecular motion of water. The tendency of ADC values in the medullary zone was quite different among the three subclasses of LN patients. ADC values in type-V patients gradually declined in the renal medulla. The shape of ADC values in the renal parenchyma was similar with that in healthy volunteers. We speculate that the main reason for this phenomenon may to maintain an increasing osmolality in the renal-medullary zone. In physiological conditions, kidneys should maintain a continuously ascending osmolality for urine concentration. In renal outer medulla, the thick ascending limb can prevent water reabsorption due to low-water permeability in the tubular wall. Although the descending limb is highly permeable to water, the direction of water movement is primarily from the inner tubular to outer tubular region due to aquaporin-1 influences. Moreover, water permeability of the inner-medullary collecting duct is also low. When vasopressin exerts influence on the water-channel aquaporin-2, water permeability of the collecting-duct wall increases. Subsequently, water can then be reabsorbed along the direction from the inner duct to the outer duct. This may explain why lower molecular motion of water is found in the medulla. However, the normal microstructure and physiological functions will be destroyed or absent in the face of tubular-interstitial injuries. Neither the loop of Henle nor the collecting duct would be able to maintain the low-water permeability under the conditions of damaged tissue. This may account for why elevated ADC values were detected in the medullary zone. Furthermore, higher renal-medullary ADC values in type-IV-subclass patients may be attributed to more serious tubular-interstitial pathological injuries. Our study also found renal ADC configuration difference in type-IV subclass patients. In superficial renal parenchyma, lower ADC values were observed in patient with impaired GFR. Since impaired GFR reflected glomerular pathological injuries, we speculated that proliferation changes in cortical glomerular encumbered water molecular motion. Meanwhile, patients with impaired GFR usually coexisted tubular-intestinal histological changes, which may damage the integrity of tubular construction. Impaired tubular construction may give raise to increased ability of water molecular motion. Based on these possibility hypotheses, the intersection structure of renal ADC values in type-IV subclass patients could be explained rationally.

Our study still had some inherent limitations. First, there are no corresponding studies of renal pathological changes and local regional ADC values distributions available in the present literature. As such, the renal-parenchyma locations of ROIs for our ADC-value measurements did not rigorously match the biopsy sites. This potential intrinsic bias may influence the reliability of our study. Second, the heterogeneity of ADC values in the renal parenchyma was easily found in our LN patients. Even in healthy volunteers, markedly lower ADC values were sporadically distributed in renal tissues. Since our study did not include functional MRI (fMRI) data such as blood-oxygen level differences (BOLDs) or diffusion-tensor imaging (DTI), we did not investigate this matter further. Third, the sample size in our current study was relatively small, which might influence the applicability of our conclusions.

## Conclusions

We conclude that DWI is a relatively simple and noninvasive clinical tool for the detection of renal pathophysiological conditions. Analyzing the configuration of renal-tissue ADC values may help to better elucidate and clinically treat the underlying pathophysiology of LN.

## Supplementary information

**Additional file 1 Figure Supplementary**: Renal ADC configuration in LN patients with type-IV subclass.

**Additional file 2 Supplementary Table 1.** Detailed information of subjects in healthy volunteers’ group.

**Additional file 3 Supplementary Table 2.** Detailed information of subjects in lupus nephritis group.

## Data Availability

The datasets used and/or analyzed during the current study are available from the corresponding author on reasonable request.

## Abbreviations

ADC: Apparent-diffusion coefficient
BOLD: Blood oxygen level dependent
CKD-EPI: Chronic Kidney Disease Epidemiology Collaboration
DWI: Diffusion-weighted imaging
DTI: Diffusion-tensor imaging
ESRD: End-stage renal disease
FOV: Field of view
fMRI: Functional magnetic resonance imaging
LN: Lupus nephritis
MRI: Magnetic resonance imaging
ROI: Region of interest
RM-ANOVA: Repeated-measures analysis of variance
SLE: Systemic lupus erythematosus
SLEDAI: SLE Disease Activity Index
SPGR: Spoiled gradient recalled echo
99mTcDPTA: Technetium-99 m diethylenetriaminepentaacetic acid
TR: Repetition time
TE: Echo time
