# Supplementary figures and images for: Detection of water-molecular-motion configuration in patients with lupus nephritis: a primary study using diffusion-weighted imaging
Source: BMC Nephrol. 2020 Jul 29;21:313. doi: 10.1186/s12882-020-01955-x (PMC7392731; doi:10.1186/s12882-020-01955-x)

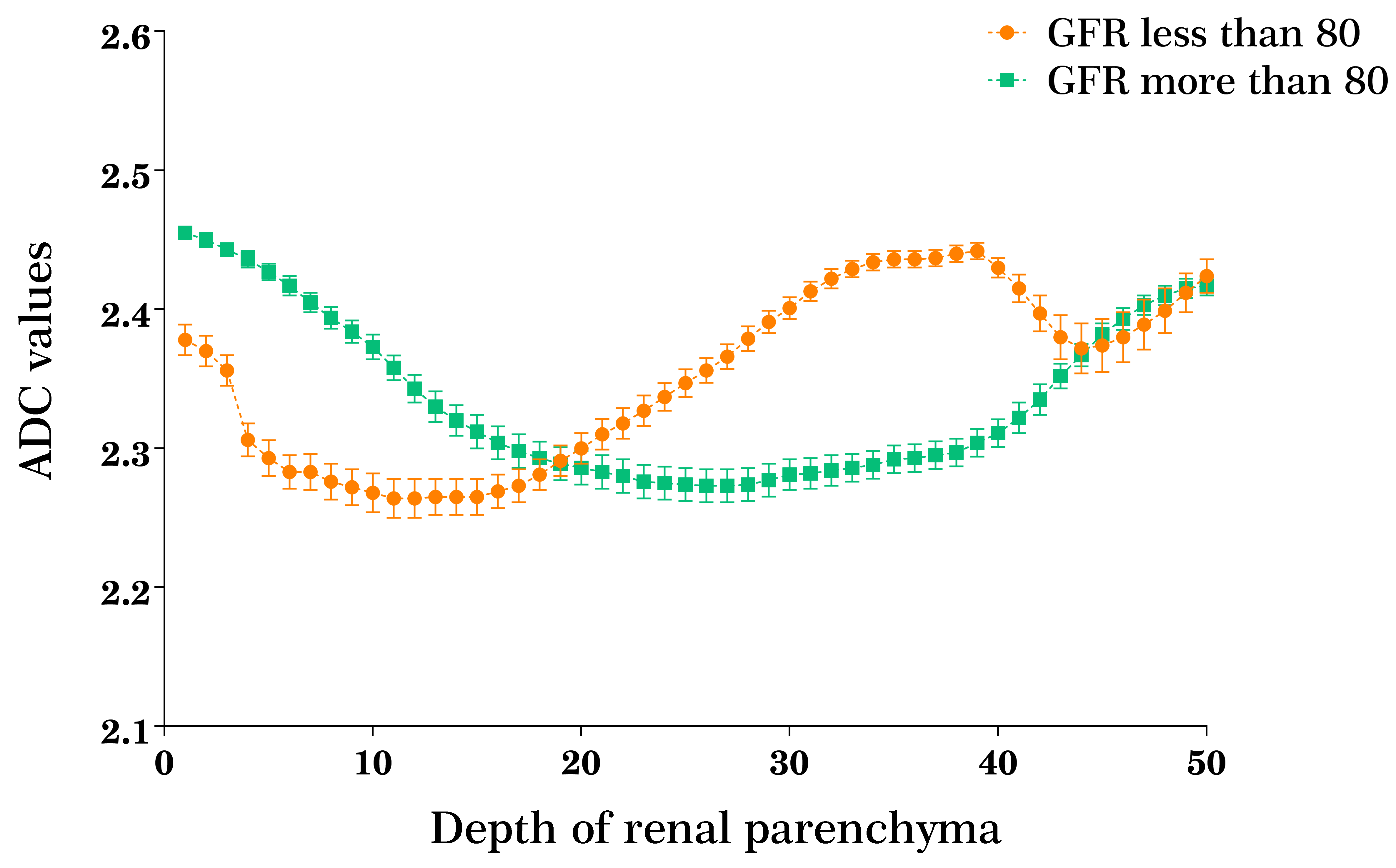

Supplement: Supplementary file 1 — Additional file 1 Figure Supplementary: Renal ADC configuration in LN patients with type-IV subclass. [file 12882_2020_1955_MOESM1_ESM.tif]
