# Supplementary material for: Detection of water-molecular-motion configuration in patients with lupus nephritis: a primary study using diffusion-weighted imaging
Source: BMC Nephrol. 2020 Jul 29;21:313. doi: 10.1186/s12882-020-01955-x (PMC7392731; doi:10.1186/s12882-020-01955-x)
Supplement: Supplementary file 2 — Additional file 2 Supplementary Table 1. Detailed information of subjects in healthy volunteers’ group. [file 12882_2020_1955_MOESM2_ESM.docx]

Table 1: Detailed information of subjects in healthy volunteers’ group.

| Case number | Age hierarchy | eGFR (ml/min·1.73m^2^) | Division of hospital | Diagnosis of disease |
| --- | --- | --- | --- | --- |
| 1 | D | 103 | Physical examination | None |
| 2 | B | 105 | Physical examination | None |
| 3 | D | 110 | Physical examination | None |
| 4 | C | 120 | Physical examination | None |
| 5 | B | 130 | Physical examination | None |
| 6 | A | 131 | Physical examination | None |
| 7 | B | 120 | General surgery | inguinal hernia |
| 8 | B | 141 | General surgery | phlebothrombosis |
| 9 | C | 99 | Endocrinology | diabetes mellitus |
| 10 | D | 99 | Ophthalmology | cataract |
| 11 | C | 116 | Dermatology | urticaria |

Note: eGFR (estimated glomerular filtration rate); Age hierarchy (A:<20; B:21~30; C:31~40; D:41~50;F:51~60; G:>61)
