# Supplementary material for: Detection of water-molecular-motion configuration in patients with lupus nephritis: a primary study using diffusion-weighted imaging
Source: BMC Nephrol. 2020 Jul 29;21:313. doi: 10.1186/s12882-020-01955-x (PMC7392731; doi:10.1186/s12882-020-01955-x)
Supplement: Supplementary file 3 — Additional file 3 Supplementary Table 2. Detailed information of subjects in lupus nephritis group. [file 12882_2020_1955_MOESM3_ESM.docx]

Table 2: Detailed information of subjects in lupus nephritis group.

| Case number | Age hierarchy | eGFR (ml/min·1.73m^2^) | PRO  (g/24h) | Pathological pattern |
| --- | --- | --- | --- | --- |
| 1 | F | 98 | 2.14 | IV-G(A/C)+V |
| 2 | C | 138 | 6.44 | V |
| 3 | C | 62 | 4.76 | IV-G(A/C)+V |
| 4 | D | 71 | 9.47 | IV-S(A/C)+V |
| 5 | A | 39 | 1.87 | IV-S(A/C) |
| 6 | B | 113 | 1.15 | III-(A/C)+V |
| 7 | A | 120 | 0.81 | III-(A/C) |
| 8 | C | 85 | 3.65 | IV-G(A/C) |
| 9 | B | 123 | 0.58 | IV-G(A/C)+V |
| 10 | D | 89 | 5.81 | IV-S(A) |
| 11 | B | 138 | 1.57 | V |
| 12 | B | 118 | 1.34 | V |
| 13 | A | 105 | 1.46 | IV-G(A/C) |
| 14 | G | 85 | 5.20 | V |
| 15 | C | 108 | 7.14 | III-(A/C)+V |
| 16 | F | 82 | 2.08 | IV-G(A/C)+V |
| 17 | C | 80 | 2.08 | III-(A/C) |
| 18 | F | 97 | 8.25 | IV-G(A/C)+V |
| 19 | B | 124 | 6.51 | III-(A/C)+V |
| 20 | D | 113 | 4.67 | IV-G(A/C) |

Note: eGFR (estimated glomerular filtration rate); PRO (proteinuria); Age hierarchy (A:<20; B:21~30; C:31~40; D:41~50;F:51~60; G:>61)
